# Supplementary material for: Macrophage-Targeted Dextran Sulfate-Dexamethasone Conjugate Micelles for Effective Treatment of Rheumatoid Arthritis
Source: Molecules. 2023 Jan 6;28(2):591. doi: 10.3390/molecules28020591 (PMC9863669; doi:10.3390/molecules28020591)
Supplement: Supplementary file 1 [file molecules-28-00591-s001.zip › molecules-2103166-supplementary.pdf]

Supporting Information

Article

Macrophage-Targeted Dextran Sulfate-dexamethasone Conjugate Micelles for Effective Treatment of Rheumatoid Arthritis

Jiangfan Han [1,2,†], Ren Na [3,†], Ningning Zhao [2,†], Xiaofeng Yuan [2], Linke Fu [2], Jianmei Jing [4], Airong Qian [1,*] and Weiliang Ye [2,*]

1 Lab for Bone Metabolism, Key Lab for Space Biosciences and Biotechnology, School of Life Sciences, Northwestern Polytechnical University, Xi'an 710072, China; jfanhan@mail.nwpu.edu.cn (J.H.)
2 Department of Pharmaceutics, School of Pharmacy, Fourth Military Medical University, Xi'an 710032, China; 18729517815@163.com (N.Z.); xiaofeng012345@163.com (X.Y.); f2314280762@163.com (L.F.)
3 Department of Epidemiology and Health Statistics, Faculty of Military Preventive Medicine, Fourth Military Medical University, Xi'an 710032, China; narentaiyang@126.com
4 Department of Radiology, Xijing Hospital, Fourth Military Medical University, Xi'an 710038, China; jjm13468927661@163.com
* Correspondence: qianair@nwpu.edu.cn (A.Q.); yaojixue@fmmu.edu.cn (W.Y.); Tel.: +86-29-8477-6783 (W.Y.)
† These authors contributed equally to this work.

Citation: Han, J.; Na, R.; Zhao, N.; Yuan, X.; Fu, L.; Jing, J.; Qian, A.; Ye, W. Macrophage-Targeted Dextran Sulfate-Dexamethasone Conjugate Micelles for Effective Treatment of Rheumatoid Arthritis. *Molecules* **2023**, *28*, x. https://doi.org/10.3390/molecules28020591

Academic Editor: Yi Lu

Received: 1 December 2022
Revised: 28 December 2022
Accepted: 29 December 2022
Published: 6 January 2023

Copyright: © 2023 by the authors. Submitted for possible open access publication under the terms and conditions of the Creative Commons Attribution (CC BY) license (https://creativecommons.org/licenses/by/4.0/).

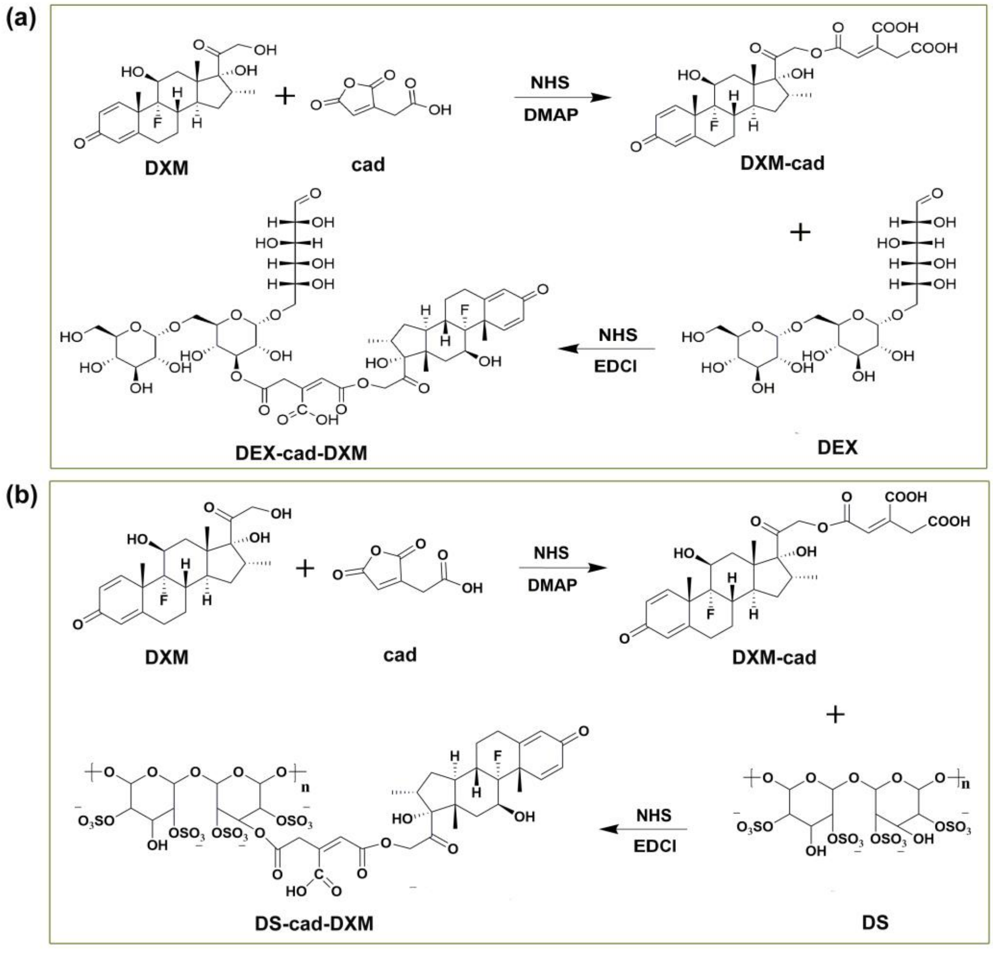

Figure S1. Synthesis route of DEX-cad-DXM (**a**) and DS-cad-DXM (**b**) conjugates.

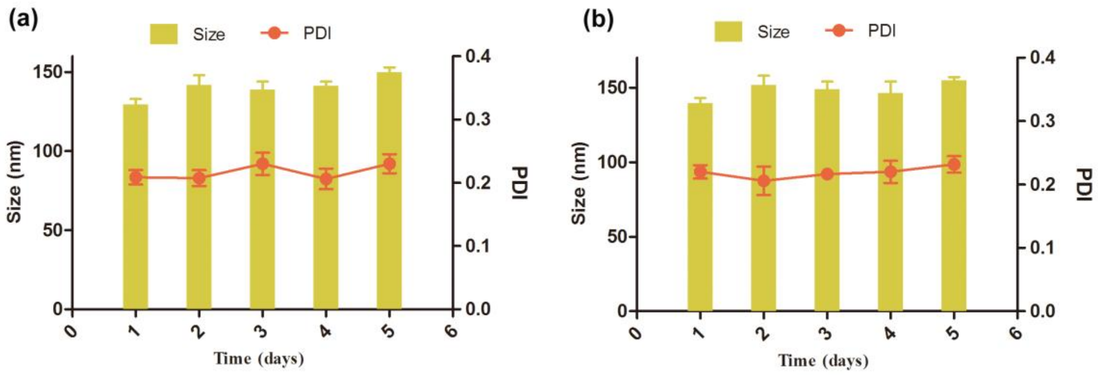

Figure S2. The stability of DXM@DS-cad-DXM micelles (**a**) and DXM@DEX-cad-DXM micelles (**b**) in pH 7.4 solution at 37 °C for 5 days. Date are presented as the mean ± SD (n = 3).

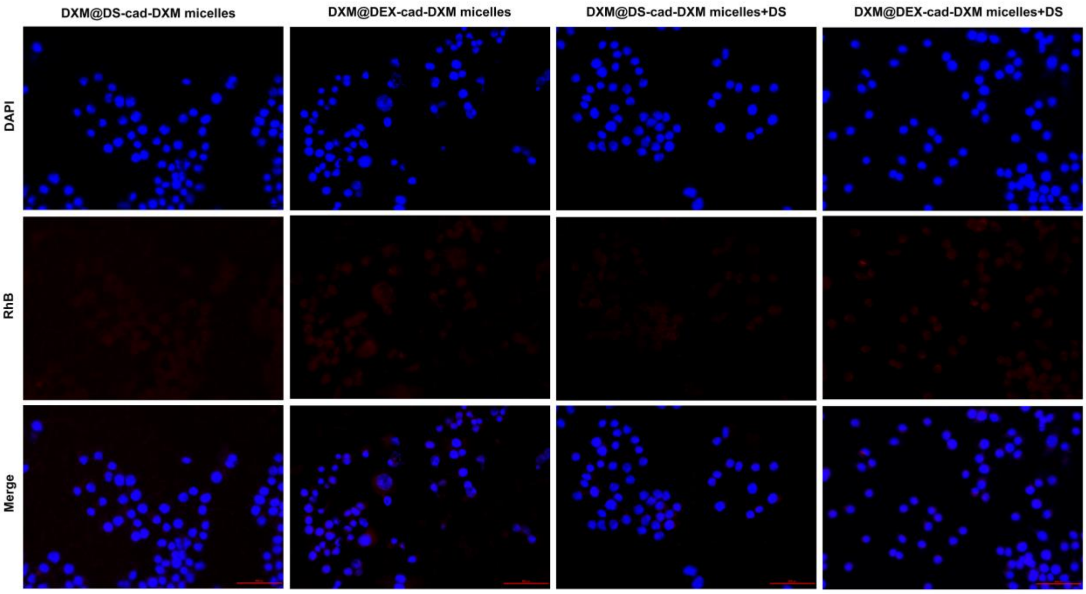

Figure S3. Fluorescent images of the DXM@DS-cad-DXM micelles and DXM@DEX-cad-DXM micelles uptaked by the unactivated RAW264.7 cells for 2 h incubation. Scale bar = 50 μm.

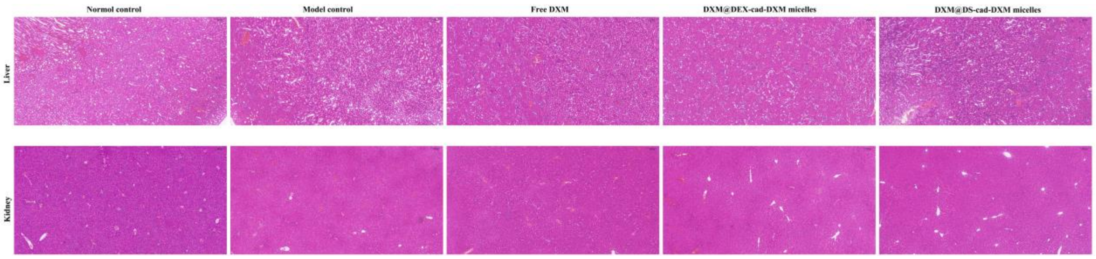

Figure S4. HE staining of liver and kidney after the treatment. Scale bar =200 μm.
